# Supplementary material for: Mild Hyperthermia-Induced Thermogenesis in the Endoplasmic Reticulum Defines Stress Response Mechanisms
Source: Cells. 2024 Jul 3;13(13):1141. doi: 10.3390/cells13131141 (PMC11240596; doi:10.3390/cells13131141)
Supplement: Supplementary file 1 [file cells-13-01141-s001.zip › cells-3068778-supplementary.pdf]

Supplement Material

Mild Hyperthermia-Induced Thermogenesis in the Endoplasmic Reticulum Defines Stress Response Mechanisms

Barbara Dukic [1,2], Zsófia Ruppert [1,3], Melinda E. Tóth [1], Ákos Hunya [1], Ágnes Czibula [4,5], Péter Bíró [6], Ádám Tiszlavicz [1], Mária Péter [1], Gábor Balogh [1], Miklós Erdélyi [6], Gyula Timinszky [4], László Vígh [1], Imre Gombos [1] and Zsolt Török [1,*]

[1] Laboratory of Molecular Stress Biology, Institute of Biochemistry, HUN-REN Biological Research Centre, 6726 Szeged, Hungary; dukic.barbara@brc.hu (B.D.); vigh.laszlo@brc.hu (L.V.); gombos.imre@brc.hu (I.G.)

[2] Doctoral School of Environmental Sciences, Faculty of Science and Informatics, University of Szeged, 6720 Szeged, Hungary

[3] Doctoral School of Biology, Faculty of Science and Informatics, University of Szeged, 6720 Szeged, Hungary

[4] Laboratory of DNA Damage and Nuclear Dynamics, Institute of Genetics, HUN-REN Biological Research Centre, 6726 Szeged, Hungary

[5] Department of Immunology, University of Szeged, 6720 Szeged, Hungary

[6] Department of Optics and Quantum Electronics, University of Szeged, 6720 Szeged, Hungary

* Correspondence: torok.zsolt@brc.hu

MEF	37°C	39°C	40°C	41°C	42°C	43°C	44°C
Hsp25	100	215.348	3530.17	30443.7	44882.2	5623.25	6370.49
Hsp60	100	116.473	151.747	178.18	148.11	86.8541	96.1483
Hsp70	100	987.194	17026.8	64359.1	94445.2	30163.6	33546.1
Hsp90aa1	100	143.396	192.964	288.786	265.737	97.716	95.705
Hsp90ab1	100	110.957	129.984	119.748	116.205	108.423	108.925
Hsf1	100	84.6745	92.7659	76.8438	82.169	91.5945	79.1869
Ire1a	100	106.93	102.693	101.396	104.247	105.214	103.526
Ire1b	100	102.811	114.472	104.729	104.729	110.191	105.458
Xbp1_total	100	131.039	127.603	94.8246	63.2878	99.7692	99.0801
Xbp1_us	100	120.025	145.229	109.682	51.0506	30.2848	31.9377
Xbp1_s	100	67.8694	44.7254	29.6787	45.5072	135.426	138.591
Bip	100	124.258	127.899	104.488	81.0378	93.088	93.7354
Atf6	100	93.9523	116.339	89.0899	84.0896	123.114	130.435
Perk	100	101.63	106.807	97.716	97.716	105.214	105.458
Chop	100	102.337	91.4888	67.6737	87.0551	64.3197	53.8369
Grp94	100	106.437	119.886	100.463	96.1483	99.3092	106.437
Atf4	100	43.4271	39.3199	28.5851	23.0579	99.3092	92.445

U2OS	37°C	39°C	40°C	41°C	42°C	43°C	44°C
HSP27	100	113.158	143.23	199.538	246.514	129.235	140.932
HSP60	100	102.456	115.535	155.473	233.216	121.982	165.29
HSP70	100	295.878	703.719	1163.18	2013.55	1758.98	2138.21
HSP90aa1	100	99.8845	119.61	141.421	185.104	107.923	119.61
HSP90ab1	100	113.945	125.266	142.076	147.597	118.646	133.947
HSF1	100	179.212	203.967	221.914	180.042	133.793	153.865
IRE1a	100	74.4839	87.1557	119.196	128.194	72.6986	85.362
XBP1_total	100	148.624	164.148	173.708	153.51	87.0551	101.983
XBP1_us	100	138.191	141.748	138.191	117.283	9.06636	26.4255
XBP1_s	100	201.391	293.494	262.685	334.808	1024.37	873.415
BIP	100	92.7659	99.1946	99.539	95.5945	93.088	94.2785
ATF6	100	85.362	101.748	86.0551	95.1538	90.5425	93.6272
PERK	100	128.491	147.257	144.727	138.351	94.3874	115.003
CHOP	100	372.782	406.992	349.026	279.917	69.7372	104.85
ATF4	100	92.445	99.539	89.9171	88.0666	102.337	104.972

Supplementary Table S1. Transcription profile of important HSP and UPR genes in MEF and U2OS cells in response to heat treatment at different temperatures (37°C, 39°C, 40°C, 41°C, 42°C, 43°C, 44°C) for one hour. Expression of target genes in treated cells was compared to non-treated control cells kept at optimal growing temperature of 37°C (results are given as a percentage, where non-treated cells = 100%). Student's t-test was used for statistical comparisons ($p<0.05$; significant change indicated with blue).

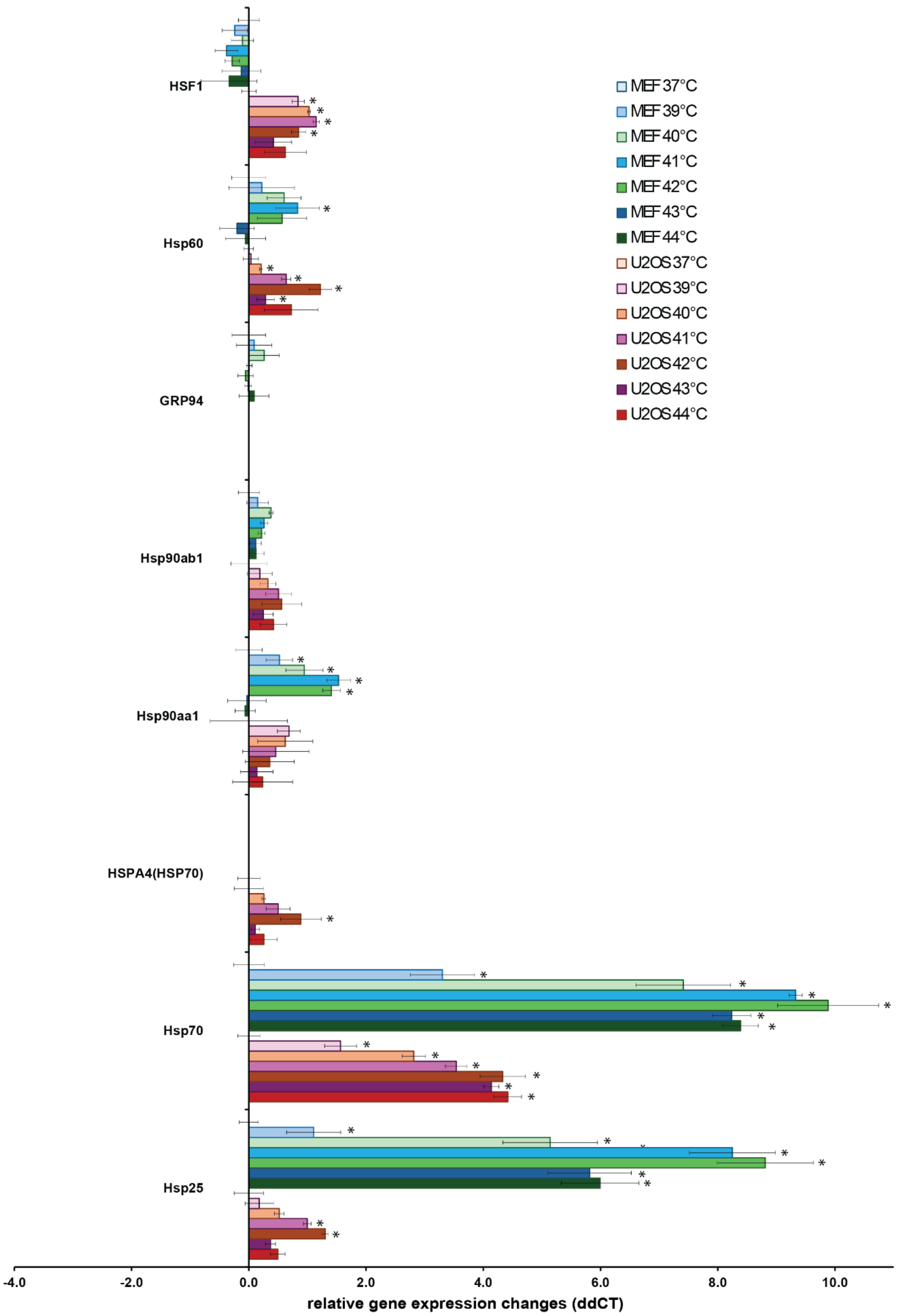
HSF1
Hsp60
GRP94
Hsp90ab1
Hsp90aa1
HSPA4(HSP70)
Hsp70
Hsp25
MEF 37°C
MEF 39°C
MEF 40°C
MEF 41°C
MEF 42°C
MEF 43°C
MEF 44°C
U2OS 37°C
U2OS 39°C
U2OS 40°C
U2OS 41°C
U2OS 42°C
U2OS 43°C
U2OS 44°C
-4.0
-2.0
0.0
2.0
4.0
6.0
8.0
10.0
relative gene expression changes (ddCT)

Supplementary Figure S1. Relative heat-shock protein (HSP) gene expression changes (ddCT) in MEF and U2OS cells in response to heat treatment at different temperatures (39°C, 40°C, 41°C, 42°C, 43°C, 44°C) for one hour. The relative expression of different HSP genes was studied using RT-qPCR (n = 3 biological repetitions). The expression of target genes in treated cells was compared to non-treated control cells kept at the optimal growing temperature of 37°C. Student's t-test was used for statistical comparisons (*p<0.05).

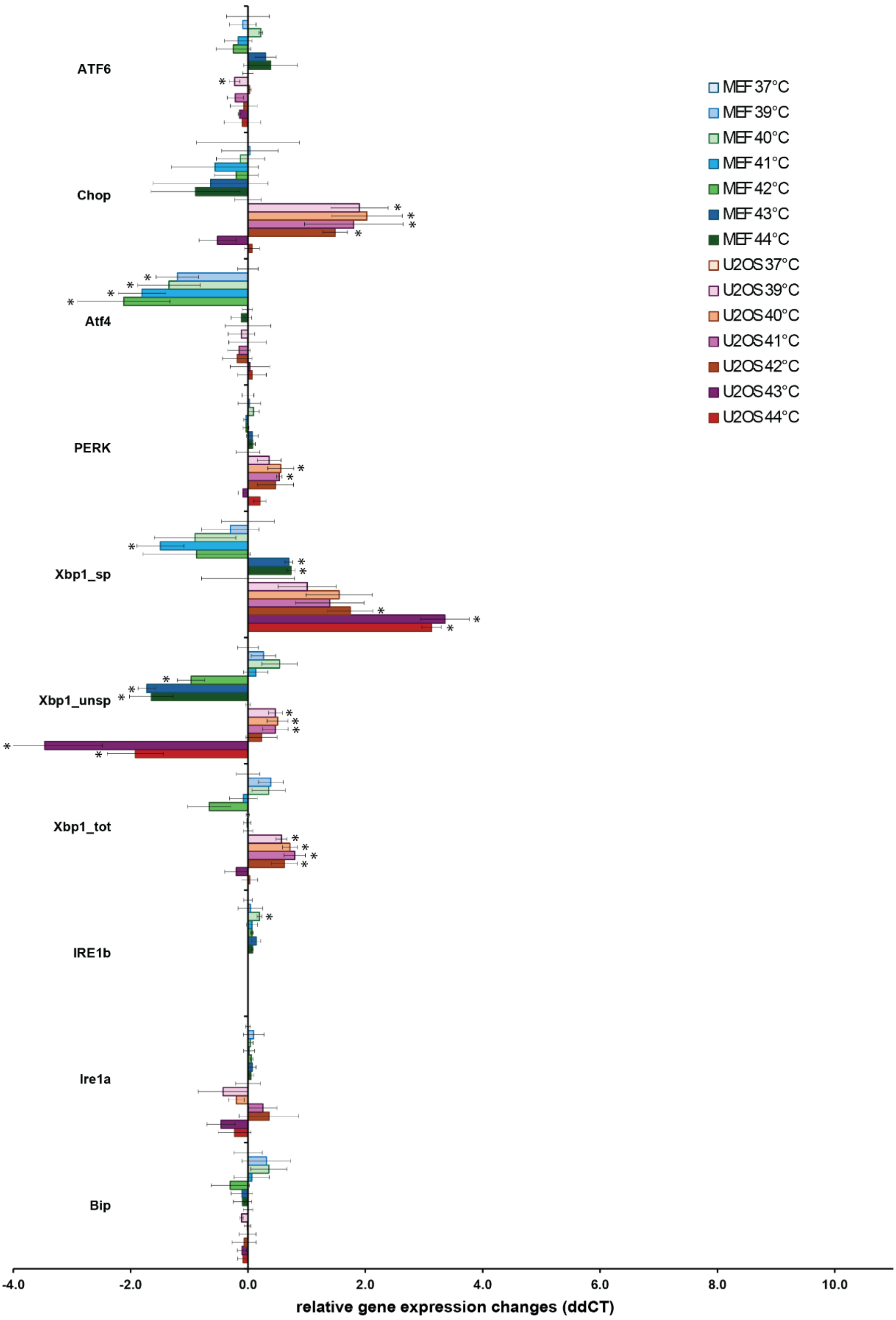

Supplementary Figure S2. Relative unfolded protein response (UPR) gene expression changes (ddCT) in MEF and U2OS cells in response to heat treatment at different temperatures (39°C, 40°C, 41°C, 42°C, 43°C, 44°C) for one hour. The relative expression of different UPR genes was studied using RT-qPCR (n = 3 biological repetitions). The expression of target genes in treated cells was compared to non-treated control cells kept at the optimal growing temperature of 37°C. Student's t-test was used for statistical comparisons (*p<0.05).

MEF

	Hsp25	Hsp70	Hsp90aa1	Hsp90ab1	Hsp60	HSF1	Bip	Ire1a	Xbp1_tot	Xbp1_us	Xbp1_sp	PERK	Atf4	Chop	ATF6
Hsp25	1.000	0.961	0.597	0.466	0.395	-0.274	-0.309	-0.006	-0.523	-0.360	-0.305	-0.048	-0.476	-0.326	-0.085
Hsp70	0.961	1.000	0.490	0.483	0.337	-0.297	-0.247	0.044	-0.409	-0.409	-0.229	0.006	-0.418	-0.342	0.049
Hsp90aa1	0.597	0.490	1.000	0.636	0.802	-0.123	-0.045	-0.045	-0.354	0.406	-0.823	-0.201	-0.906	-0.067	-0.465
Hsp90ab1	0.466	0.483	0.636	1.000	0.752	0.241	0.318	-0.154	0.079	0.307	-0.387	0.311	-0.496	-0.157	0.111
Hsp60	0.395	0.337	0.802	0.752	1.000	0.276	0.019	-0.348	-0.265	0.439	-0.727	-0.011	-0.735	-0.159	-0.209
HSF1	-0.274	-0.297	-0.123	0.241	0.276	1.000	-0.107	-0.185	0.064	0.102	0.091	0.251	0.147	-0.278	0.193
Bip	-0.309	-0.247	-0.045	0.318	0.019	-0.107	1.000	-0.081	0.766	0.603	0.047	0.350	0.179	0.513	-0.013
Ire1a	-0.006	0.044	-0.045	-0.154	-0.348	-0.185	-0.081	1.000	0.126	-0.138	0.166	0.059	-0.019	0.049	-0.054
Xbp1_tot	-0.523	-0.409	-0.354	0.079	-0.265	0.064	0.766	0.126	1.000	0.451	0.285	0.322	0.387	0.319	0.245
Xbp1_us	-0.360	-0.409	0.406	0.307	0.439	0.102	0.603	-0.138	0.451	1.000	-0.599	-0.113	-0.366	0.421	-0.399
Xbp1_sp	-0.305	-0.229	-0.823	-0.387	-0.727	0.091	0.047	0.166	0.285	-0.599	1.000	0.517	0.830	-0.040	0.503
PERK	-0.048	0.006	-0.201	0.311	-0.011	0.251	0.350	0.059	0.322	-0.113	0.517	1.000	0.343	-0.143	0.267
Atf4	-0.476	-0.418	-0.906	-0.496	-0.735	0.147	0.179	-0.019	0.387	-0.366	0.830	0.343	1.000	0.030	0.466
Chop	-0.326	-0.342	-0.067	-0.157	-0.159	-0.278	0.513	0.049	0.319	0.421	-0.040	-0.143	0.030	1.000	-0.253
ATF6	-0.085	0.049	-0.465	0.111	-0.209	0.193	-0.013	-0.054	0.245	-0.399	0.503	0.267	0.466	-0.253	1.000

U2OS

	Hsp25	Hsp70	Hsp90aa1	Hsp90ab1	Hsp60	HSF1	Bip	Ire1a	Xbp1_tot	Xbp1_unsp	Xbp1_sp	PERK	Atf4	Chop	ATF6
Hsp25	1.000	0.650	0.096	0.537	0.816	0.590	0.009	0.464	0.505	0.200	0.158	0.545	-0.369	0.402	-0.040
Hsp70	0.650	1.000	0.066	0.643	0.723	0.451	-0.101	0.156	0.058	-0.419	0.790	0.197	0.063	-0.052	-0.053
Hsp90aa1	0.096	0.066	1.000	0.402	-0.014	0.425	0.450	0.111	0.536	0.321	-0.024	0.260	0.397	0.287	0.188
Hsp90ab1	0.537	0.643	0.402	1.000	0.718	0.609	0.525	0.668	0.431	0.151	0.306	0.481	0.401	0.137	0.351
Hsp60	0.816	0.723	-0.014	0.718	1.000	0.450	0.049	0.552	0.215	0.044	0.298	0.378	-0.087	0.091	0.135
HSF1	0.590	0.451	0.425	0.609	0.450	1.000	0.107	0.287	0.724	0.468	0.052	0.743	-0.103	0.719	-0.009
Bip	0.009	-0.101	0.450	0.525	0.049	0.107	1.000	0.547	0.362	0.298	-0.201	0.254	0.554	-0.036	0.632
Ire1a	0.464	0.156	0.111	0.668	0.552	0.287	0.547	1.000	0.397	0.465	-0.306	0.452	0.169	0.145	0.355
Xbp1_tot	0.505	0.058	0.536	0.431	0.215	0.724	0.362	0.397	1.000	0.749	-0.294	0.805	-0.094	0.841	0.052
Xbp1_unsp	0.200	-0.419	0.321	0.151	0.044	0.468	0.298	0.465	0.749	1.000	-0.759	0.598	-0.142	0.758	0.153
Xbp1_sp	0.158	0.790	-0.024	0.306	0.298	0.052	-0.201	-0.306	-0.294	-0.759	1.000	-0.219	0.257	-0.323	-0.101
PERK	0.545	0.197	0.260	0.481	0.378	0.743	0.254	0.452	0.805	0.598	-0.219	1.000	-0.295	0.753	0.086
Atf4	-0.369	0.063	0.397	0.401	-0.087	-0.103	0.554	0.169	-0.094	-0.142	0.257	-0.295	1.000	-0.356	0.527
Chop	0.402	-0.052	0.287	0.137	0.091	0.719	-0.036	0.145	0.841	0.758	-0.323	0.753	-0.356	1.000	-0.137
ATF6	-0.040	-0.053	0.188	0.351	0.135	-0.009	0.632	0.355	0.052	0.153	-0.101	0.086	0.527	-0.137	1.000

Supplementary Table S2. Heat map representation of correlation data between the heat-shock protein (HSP) and unfolded protein response (UPR) genes of MEF and U2OS cells following exposure to heat. Pearson correlation analysis was conducted to examine the relationship between the induced HSP and UPR genes. Color code: -1 = total negative linear correlation (green); 0 = no correlation (yellow); +1 = total positive correlation (red).

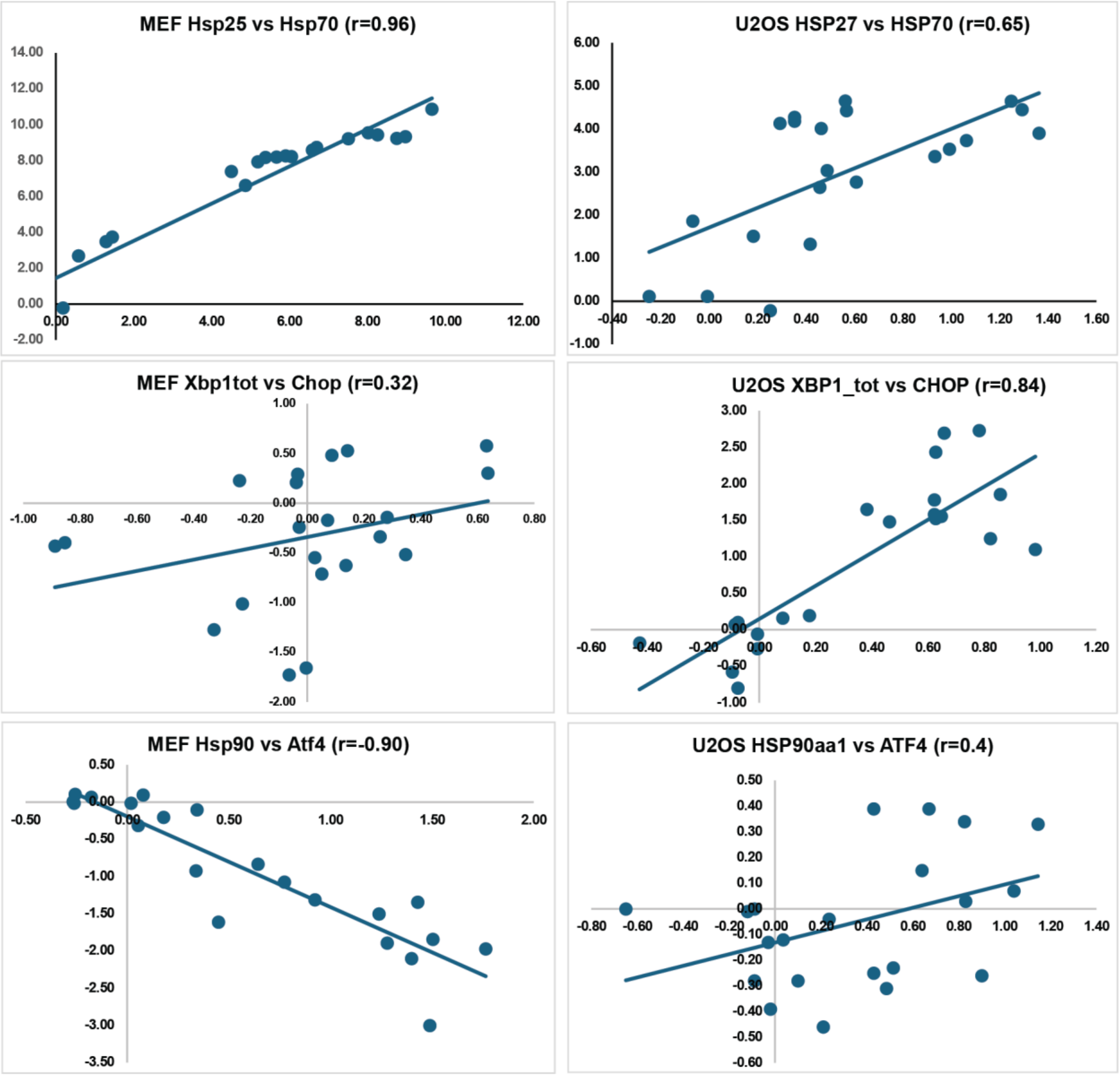

Supplementary Figure S3. Correlation between HSR and UPR genes in MEF and U2OS cells. Circles in scatter plots represent individual samples; n = 3 (independent biological replicates) at each applied temperatures from 37 to 44°C. Solid lines show linear regressions, r values represent Pearson correlation coefficients.

Mouse gene		forward primer	reverse primer
1	*Gapdh*	GGGTTCCTATAAATACGGACTGC	CCATTTTGTCTACGGGACGA
2	*Xbp1_total*	TGGCCGGGTCTGCTGAGTCCG	GTCCATGGGAAGATGTTCTGG
3	*Xbp1_us*	CAGCACTCAGACTATGTGCA	GTCCATGGGAAGATGTTCTGG
4	*Xbp1_s*	CTGAGTCCGAATCAGGTGCAG	GTCCATGGGAAGATGTTCTGG
5	*Bip*	TTCAGCCAATTATCAGCAAACTCT	TTTTCTGATGTATCCTCTTCACCAGT
6	*Ire1a/Ern1*	CTGGCTTCTCATAGGACACCAT	TCTCGATGTTTGGGCAGGTT
7	*Ire1b/Ern2*	CCATGAGGAACAAGAAGCACC	AGGAAGTTGGCCTAGTGTCTG
8	*Perk*	TCCTGCTTTGCATCGTAGCC	CAGACTCCTTCCGCTGCCTG
9	*Atf4*	CTAAGCCATGGCGCTCTTCA	GCTGGATTCGAGGAATGTGC
10	*Atf6*	CCAGATGAAGACTGGGAGTCG	GCTGCATCAAAGTGCACATCA
11	*Chop*	CCACCACACCTGAAAGCAGAA	AGGTGAAAGGCAGGGACTCA
12	*Hsf1*	GGGAAACAGGAGTGTATGGACT	CTTGTTGACAACTTTTTGCTGCT
13	*Hsp25*	ATCCCCTGAGGGCACACTTA	GGAATGGTGATCTCCGCTGAC
14	*Hsp60*	CACAGTCCTTCGCCAGATGAG	CTACACCTTGAAGCATTAAGGCT
15	*Hsp70*	GAGATCGACTCTCTGTTCGAGG	GCCCGTTGAAGAAGTCCTG
16	*Hsp90aa1*	GACGCTCTGGATAAAATCCGTT	TGGGAATGAGATTGATGTGCAG
17	*Hsp90ab1*	AAACAAGGAGATTTTCCTCCGC	CCGTCAGGCTCTCATATCGAAT
**Human gene**		**forward primer**	**reverse primer**
18	*GAPDH*	ACAACTTTGGTATCGTGGAAGG	GCCATCACGCCACAGTTTC
19	*RPL27*	CGCAAAGCTGTCATCGTG	GTCACTTTGCGGGGGTAG
20	*XBP1_total*	TGAAAAACAGAGTAGCAGCTCAGA	CCCAAGCGCTGTCTTAACTC
21	*XBP1_us*	CAGACTACGTGCACCTCTGC	CTGGGTCCAAGTTGTCCAGAAT
22	*XBP1_s*	GCTGAGTCCGCAGCAGGT	CTGGGTCCAAGTTGTCCAGAAT
23	*BIP*	GACCACCTACTCCTGCGTC	GTGAAGGCGACATAGGACGG
24	*IRE1a/ERN1*	AATTGTGTACCGGGGCATGT	TCACGGTCTGCGAAGCTAAA
25	*IRE1b/ERN2*	CGCTGAGTCCACAGGTTCATA	GTCTGCTTGCTTAGTGCGTG
26	*PERK*	GAGAGCAGGAGCCTCGG	GATAATTACTAATGACCTGCCGC
27	*ATF4*	TTAAGCCATGGCGCTTCTCA	GCTGGAATCGAGGAATGTGCT
28	*ATF6*	ACCCGTATTCTTCAGGGTGC	TCCCTGAGTTCCTGCTGATAC
29	*CHOP*	ACCTGAGGAGAGAGTGTTCA	TCAGTCTGGAAAAGCACATCT
30	*HSF1*	AGCCTGGCCAGTATCCAAGA	AGCTGCTTCCCTGAATCCG
31	*HSP27*	GTCCCTGGATGTCAACCACT	GACTGGGATGGTGATCTCGT
32	*HSP60*	ACTCGGAGGCGGAAGAAAAA	GTAACCGAAGCATTTCTCGGC
33	*HSP70/HSPA1A*	CCCCACCATTGAGGAGGTAG	AGGAAATGCAAAGTCTTGAAGCTC
34	*HSP90AA1*	GAATACCCGCGCGACCGT	TTAACAGGTGCCCTGCTTCTCAG
35	*HSP90AB1*	GGGTATCGGAAAGCAAGCCT	GCACTTCCTCAGGCATCTTGAA

Supplementary Table S3. Primer sequences utilized in RT-qPCR reactions. (Mouse Xbp1 primers are from Oslowski CM, Urano F. Measuring ER stress and the unfolded protein response using mammalian tissue culture system. Methods Enzymol. 2011;490:71-92. doi: 10.1016/B978-0-12-385114-7.00004-0. PMID: 21266244; PMCID: PMC3701721.)

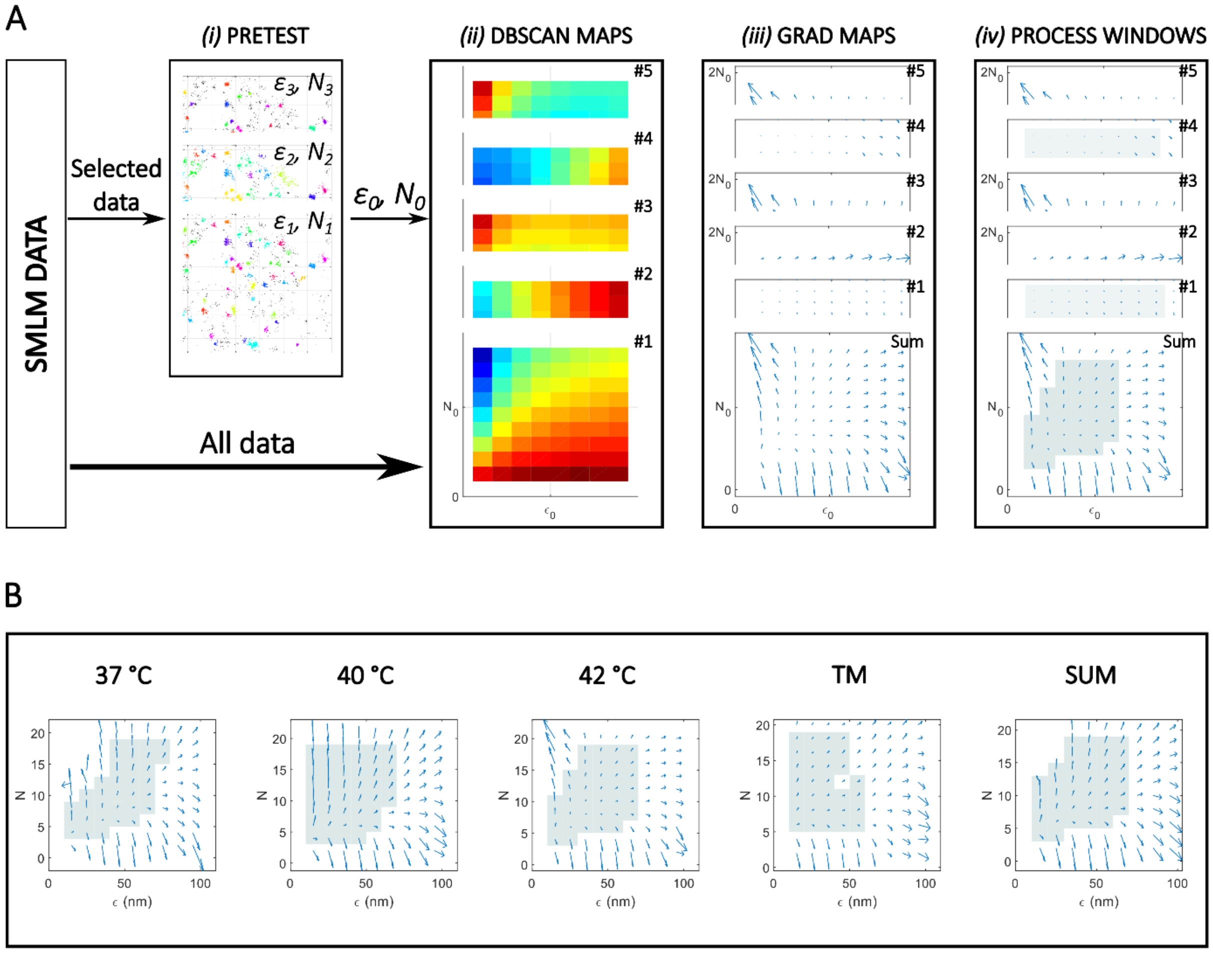

Supplementary Figure S4. Selection procedure of the optimum DBSCAN parameters. The DBSCAN cluster analyzer algorithm uses two input parameters, a distance (ε) and a minimum number of points (N) values. Ideally a slight change in such parameters do not significantly affect the feature of the final clustered images, therefore quantitative analysis can be associated with real treatments and changes in the sample. Taking into account this condition, the optimum ε and N values were selected via a four-step process (A). First, a pretest cluster analysis (i) was performed on the same type of samples with several ε and N values to visually find the optimum clustered images. In the present work ε_0=55 nm and N_0=12 were selected. Next, DBSCAN cluster analyses (ii) were performed using the parameter window with size of 2ε_0 and 2N_0, and DBSCAN maps were generated using five merit functions (cluster area mean and standard deviation, localization number per cluster mean and standard deviation and number of clusters). Next, gradient maps (iii) for all DBSCAN maps were generated. At the same time the normalized gradient map of the sum was also calculated. Finally, a process window (iv) was selected on the gradient maps, where the sum gradient value was less than 0.25. The process was repeated for all the treated, the control and the sum images (B). The optimum ε-N value was defined as the center of such process windows. Based on these results, the originally selected parameter values were slightly modified to ε=45 nm and N=10, and all the cluster analyses were performed under such conditions.
